# The impact of the COVID-19 pandemic on radiotherapy in Japan: nationwide surveys from May 2020 through June 2021

**DOI:** 10.1093/jrr/rrac055

**Published:** 2022-09-16

**Authors:** Keisuke Tamari, Yasushi Nagata, Takashi Mizowaki, Takeshi Kodaira, Hiroshi Onishi, Kazuhiko Ogawa, Yoshiyuki Shioyama, Naoyuki Shigematsu, Takashi Uno

**Affiliations:** Department of Radiation Oncology, Osaka University Graduate School of Medicine, Osaka, 565-0871, Japan; Department of Radiation Oncology, Hiroshima University Hospital, Hiroshima, 734-8551, Japan; Department of Radiation Oncology and Image-Applied Therapy, Graduate School of Medicine, Kyoto University, Kyoto, 606-8507, Japan; Department of Radiation Oncology, Aichi Cancer Center Hospital, Aichi, 464-0021, Japan; Department of Radiology, University of Yamanashi School of Medicine, Yamanashi, 409-3898, Japan; Department of Radiation Oncology, Osaka University Graduate School of Medicine, Osaka, 565-0871, Japan; Ion Beam Therapy Center, SAGA-HIMAT Foundation, Saga, 841-0071, Japan; Department of Radiology, Keio University School of Medicine, Tokyo, 160-0016, Japan; Department of Radiology, Chiba University Graduate School of Medicine, Chiba, 260-8677, Japan

**Keywords:** COVID-19, radiotherapy, questionnaire, hypofractionation

## Abstract

A longitudinal online questionnaire survey on the impact of coronavirus disease 2019 (COVID-19) on the operation of radiotherapy departments in Japan was conducted. Approximately 26.1–70.9% of the radiotherapy departments participated, and their responses were collected in May, July and November 2020, and February and June 2021. The survey results revealed that while the number of patients receiving radiotherapy decreased in 41.2% and 30.7% of institutions in May 2020 and June 2021, respectively, it increased in 4% and 16.8% of institutions in May 2020 and June 2021, respectively. There were a few institutions limiting or postponing patient treatments in June 2021. The hypofractionated regimen was used more during the pandemic than during the pre-pandemic period, particularly for the treatment of breast and prostate cancers as well as for palliation. Infection control measures for patients and staff were followed. Approximately 20% of the respondent institutions had cases of patients with COVID-19 infection receiving radiotherapy. Most institutions encountered challenges in the continuous provision of radiotherapy for patients with COVID-19. In conclusion, COVID-19 had a multifaceted impact on the operations of radiotherapy departments in Japan. Further follow-up and analysis are warranted to understand the long-term impact of COVID-19 on radiotherapy.

## INTRODUCTION

Coronavirus disease 2019 (COVID-19) is caused by severe acute respiratory syndrome *coronavirus 2* (SARS-CoV-2). SARS-CoV-2 was first identified in Wuhan, China in December 2019, but within a few months, it had spread to develop into a pandemic, disrupting the life of different societies worldwide [1]. Moreover, it substantially interfered with the normal operations of medical institutions. The Japanese Society for Radiation Oncology (JASTRO) set up a COVID-19 committee on April 21, 2020, to address the impact of the pandemic on radiotherapy and to provide treatment recommendations and up-to-date information to both radiation oncologists and patients in Japan.

We conducted our first nationwide survey on radiation oncologists in April 2020, focusing on infection control measures against COVID-19, which were lacking in some radiotherapy departments at that time. Because the number of patients with COVID-19 during the initial period of the pandemic in Japan was small, the survey revealed minimal impact of COVID-19 on the operations of radiotherapy departments [2]. However, the radiotherapy departments in other countries were adversely affected [3,4], and patients with cancer were at high risk of COVID-19-related mortality [5,6]. Therefore, JASTRO continued to conduct regular investigations on the impact of COVID-19. Japan experienced four waves of infection between April 2020 and June 2021, and JASTRO conducted five surveys in radiotherapy departments.

This study aimed to evaluate the status of radiotherapy in Japan during the COVID-19 pandemic, with a focus on patient volume changes, infection control measures, COVID-19 incidence and trends in hypofractionated regimen use, through a retrospective analysis of data collected from JASTRO surveys.

## MATERIALS AND METHODS

This study was approved by the academic committee of JASTRO. The online questionnaire was developed using Google Forms (www.google.com/forms). JASTRO conducted five surveys in radiotherapy departments in May, July and November 2020 and in February and June 2021, and a total of 545, 239, 243, 201 and 238 institutions provided responses during the corresponding time periods. The survey items mainly included patient volume, infection control measures, COVID-19 incidence and trends in hypofractionated regimen use. The first survey included 23 questions, which were revised before each subsequent survey; the fifth survey included 29 questions. The first survey was sent to the institutions through postal mail and subsequent surveys were sent through email. The responses to each survey were collected and analyzed in Excel. Data on the overall COVID-19 incidence in Japan were obtained from the open-access public health database of the Ministry of Health, Labour and Welfare [7] and used as reference for the COVID-19 incidence in radiotherapy departments.

Data analysis was performed using JMP Pro 15.1.0 (SAS Institute Inc., Cary, NC). The chi-square test was performed to determine significant changes in the characteristics of institutions, increase/decrease in the number of patients, limitations in the number of patients, postponement of radiotherapy/follow-up, infection control measures and hypofractionation regimens across the five surveys. A *P* value of < 0.05 was considered statistically significant.

## RESULTS

### Characteristics of the respondent institutions

Table 1 shows the characteristics of the respondent institutions. The number of institutions that responded to the first survey (*n* = 545) was more than twice the number that responded to all the subsequent iterations of the survey (*n* < 250). The annual number of patients receiving radiotherapy was < 200 in 25.9–37.1% of the institutions, 201–500 in 39.9–46.5%, 501–1000 in 17.2–23.5%, 1001–1500 in 1.8–3.8% and ≥ 1501 in 1.2–2.5%. In addition, 98.7–99.6% of the respondent institutions used X-ray therapy, 2.9–4.9% used proton therapy, 0–1.2% used carbon beam therapy, 24.2–33.3% used brachytherapy, 29.7–39.5% used radioisotope therapy and 2.6–4% used gamma knife radiosurgery. In each survey, the responses were collected from a group of institutions with similar patient number (*P* = 0.982) and similar types of radiotherapy (*P* = 0.986). Institutions accepting COVID-19 admissions significantly increased from 79.1% in May 2020 to 91.5% in June 2021 (*P* = 0.031). In May 2020, 81.8% of institutions had COVID-19 specialists, which increased to 91.5% in June 2021 (*P* = 0.307). In November 2020 and June 2021, 70.8% and 77.5% of institutions prohibited inpatient visits, respectively (*P* = 0.320). Moreover, 7.4% and 6% prohibited the escorting of outpatients in November 2020 and June 2021, respectively (*P* = 0.851).

**Table 1:** Characteristics of the institutions responding to each survey

	May 2020	Jul 2020	Nov 2020	Feb 2021	Jun 2021	*P* value
Number of institutions responding	545	239	243	201	238	
	Number (%) of all responding institutions					
Number of patients per institution per year						
0–200	202 (37.1)	64 (26.8)	63 (25.9)	56 (27.9)	74 (31.1)	0.982
201–500	229 (42.0)	107 (44.8)	113 (46.5)	92 (45.8)	95 (39.9)	
501–1000	94 (17.3)	56 (23.4)	55 (22.6)	43 (21.4)	56 (23.5)	
1001–1500	10 (1.8)	9 (3.8)	9 (3.7)	5 (2.5)	9 (3.8)	
>1501	10 (1.8)	3 (1.3)	3 (1.2)	5 (2.5)	4 (1.7)	
Types of radiotherapy provided						
X-ray	543 (99.6)	236 (98.7)	240 (98.8)	200 (99.5)	236 (99.2)	0.937
Proton therapy	18 (3.3)	8 (3.3)	12 (4.9)	8 (4.0)	7 (2.9)	0.954
Carbon-ion	5 (0.9)	3 (1.2)	0 (0.0)	0 (0.0)	2 (0.8)	0.547
Brachytherapy	132 (24.2)	78 (32.6)	81 (33.3)	62 (30.9)	78 (32.6)	0.604
Radioisotope therapy	162 (29.7)	90 (37.7)	96 (39.5)	71 (35.3)	88 (36.8)	0.651
GammaKnife	14 (2.6)	7 (2.9)	8 (3.3)	8 (4.0)	8 (3.3)	0.986
Measures against COVID-19 in institutions						
Admit patients with COVID-19	432 (79.1)	189 (79.1)	206 (84.8)	180 (89.6)	218 (91.5)	0.031
Employ COVID-19 experts	446 (81.8)	211 (88.3)	218 (89.7)	178 (88.6)	214 (91.5)	0.307
Prohibit visits to inpatients	NA	NA	172 (70.8)	160 (79.6)	179 (77.5)	0.320
Prohibit escorting of outpatients	NA	NA	18 (7.4)	11 (5.5)	14 (6.0)	0.851

NA, not assessed.

### Changes in the number of patients receiving radiotherapy in Japan during the COVID-19 pandemic

Changes in the number of patients receiving radiotherapy at the respondent institutions were assessed. The self-reported responses were qualitatively categorized as decreasing, having no change and increasing. The percentage of institutions with the observed category was assessed as shown in Table 2. The results of the chi-square test showed that a number of institutions observed a significant decrease in the number of patients over time (from 41.2% to 30.7%, *P* = 0.015), whereas another number of institutions observed a significant increase in the number of patients (from 4% to 16.8%, *P* = 0.015).

**Table 2:** Changes in the number of patients in radiotherapy departments during the COVID-19 pandemic

	May 2020	Jul 2020	Nov 2020	Feb 2021	Jun 2021	*P* value
	% of institutions reporting					
The number of patients receiving radiotherapy						
Decrease	41.2	36.4	25.5	27.9	30.7	0.015
No change	54.8	58.6	61.7	59.2	52.5	
Increase	4.0	5.0	12.8	12.9	16.8	
Placed limit on number of patients	16.9	5.9	2.5	4.0	0.8	0.001
Postponed treatment to patients						
None	NA	NA	97.9	96.5	98.7	0.577
Prostate cancer	NA	NA	2.0	3.0	0.8	0.500
Breast cancer	NA	NA	0.4	1.0	0.8	0.871
Postponed follow-up	43.9	33.6	25.5	27.4	19.3	0.003

NA, not assessed

Restrictions in the number of patients who could be treated at the institutions (yes/no binary responses) were evaluated as shown in Table 2. The percentage of institutions that implemented restrictions, as shown in each survey, indicates that this measure was not commonly adopted to begin with (only 17% of responding institutions in May 2020) and that over time it was almost completely disregarded (< 1% by June 2021, *P* = 0.001).

In November 2020, we started the survey on the postponement of any radiotherapy treatments in general, particularly for breast and prostate cancers. Next, binary categorical (yes/no) responses were collected, as presented in Table 2. Treatment postponement was only observed in < 4% of institutions, and this did not change during the pandemic. The majority of the postponements were for less urgent prostate cancer treatments.

Initially, the postponement of patient follow-up was practiced in 43.9% of institutions in May 2020. However, it decreased significantly over time (< 20% in June 2021, *P* = 0.003), as shown in Table 2.

### Infection control measures implemented at radiotherapy departments

Survey data collected on the implementation of various infection control measures in the responding radiotherapy departments are summarized in Table 3. Following the early stages of the pandemic, infection control measures were strengthened in each institution. Over time, a definite improvement was achieved in the infection control measures implemented and enforced in patients and staff in the radiotherapy departments. Data on body temperature measurements were collected more often at the department than at home for patients and more often at home than in the department for staff. Basic infection control measures, including wearing of masks, hand hygiene, disinfection of common items and social distancing, were implemented more frequently by both patients and staff from May 2020 onward and were further strengthened thereafter. Approximately 32.1% of staff wore personal protective equipment in May 2020, and this number significantly increased to 58.8% in June 2021 (*P* < 0.001). There was a significant increase in the number of institutions where staff consumed their meals separately (> 80% in June 2021, *P* = 0.028).

**Table 3:** Infection control measures in radiotherapy department

	May 2020	Jul 2020	Nov 2020	Feb 2021	Jun 2021	*P* value
	% of institutions reporting					
Infection control measures in patients						
Body temperature measurement at home	61.5	69	69.1	75.1	68	0.362
Body temperature measurement at department	64.4	64.4	72.4	81.1	79	0.014
Wearing mask	80.7	89.1	96.7	98	98.7	< 0.001
Hand hygiene	80.2	87.4	93.4	94.5	92.9	0.006
Keeping social distance among patients	90.1	92.9	96.3	96.5	95	0.285
History of contact with COVID-19	59.6	68.2	71.6	65.7	68.5	0.462
COVID-19 screening for patients before radiotherapy						
No screening	NA	NA	NA	47.3	45.7	0.820
Before patient admission	NA	NA	NA	35.4	51.9	0.018
For all patients	NA	NA	NA	0.8	1.6	0.600
For patients with head and neck cancer	NA	NA	NA	1.2	0.8	0.776
For patients with lung cancer	NA	NA	NA	0.4	0.4	1.000
Before operating room procedure	NA	NA	NA	0.8	0.8	1.000
When COVID-19 is suspected in patients undergoing radiotherapy,						
Test for COVID-19	NA	NA	15.6	30.8	31.5	0.012
Ask a COVID-19 expert	NA	NA	76.1	65.2	63.0	0.097
Infection control measures in RT staffs						
Body temperature measurement at home	78.9	82.4	84.8	85.6	87.4	0.537
Body temperature measurement at department	41.8	39.7	49.4	58.2	52.5	0.052
Wearing mask	98.7	99.2	99.2	99	100	0.751
Hand hygiene	98.9	99.2	98.8	100	100	0.514
Wearing PPE	32.1	37.2	52.3	47.8	58.8	< 0.001
Keeping social distance among staffs	69.0	71.1	77.8	80.6	80.3	0.182
Eating individual meals	66.9	65.1	67.9	80.6	79	0.028
Regular disinfection of common items	90.3	92.1	92.6	96	94.5	0.536
Ventilation of the radiotherapy department	75.4	78.2	80.7	75.6	81.1	0.787
Separate treatment time zones for outpatients and inpatients	28.6	32.6	31.3	35.3	29.4	0.859
Preparation of business continuity plans	23.3	30.1	34.6	32.3	32.4	0.452
Two teams of staff	12.1	8.4	7.4	6.5	5	0.436
Downsizing of conferences	55.2	45.2	40.7	46.3	41.6	0.254
Meetings and documentations of COVID-19 measures	51.6	59.8	59.7	58.7	57.5	0.757
Vaccination rate of staffs						
100%	NA	NA	NA	NA	68.9	
90–99%	NA	NA	NA	NA	26.9	
< 90%	NA	NA	NA	NA	4.2	

Abbreviations: RT, radiotherapy; PPE, personal protective equipment; NA, not assessed.

By June 2021, 51.9% of the institutions screened patients for COVID-19 prior to admission for radiotherapy, and only 1.6% of institutions screened all patients at the radiotherapy departments. Approximately 15.6% of institutions tested patients receiving radiotherapy for COVID-19 who had symptoms such as fever in November 2020, and this number significantly increased to 31.5% in June 2021 (*P* = 0.012). Furthermore, 76.1% and 63% of institutions consulted a specialized department for COVID-19 in November 2020 and June 2021, respectively (*P* = 0.097).

The two-dose vaccination rates of the radiotherapy department staff were 100%, 90–100% and < 90% in 68.9%, 26.9% and 4.2% of the institutions, respectively, in June 2021. In addition, 2.1% of the respondents themselves did not want to be vaccinated with the COVID-19 vaccine in June 2021.

The hypofractionated regimen for breast cancer (36.8–41%) and prostate cancer (16.4–27.4%) treatment as well as for palliation (24.3–35.8%) was more commonly used before the COVID-19 pandemic; however, there was no significant difference in the adoption of this regimen during the pandemic, as shown in Table 4.

**Table 4:** Percentage of institutions for which less hypofractionated schedule has been adopted more than before pandemic

	Nov 2020	Feb 2021	Jun 2021	*P* value
	% of institutions reporting			
Breast cancer	38.7	36.8	41	0.830
Palliation	24.3	35.8	33.3	0.175
Prostate cancer	19.8	16.4	27.4	0.154
Head and neck cancer	2.9	1.5	2.6	0.775
Lung cancer	2.1	2.5	1.7	0.925
Brain tumor	2.5	0.5	2.6	0.378

### COVID-19 cases in the radiotherapy department

**Fig. 1:**
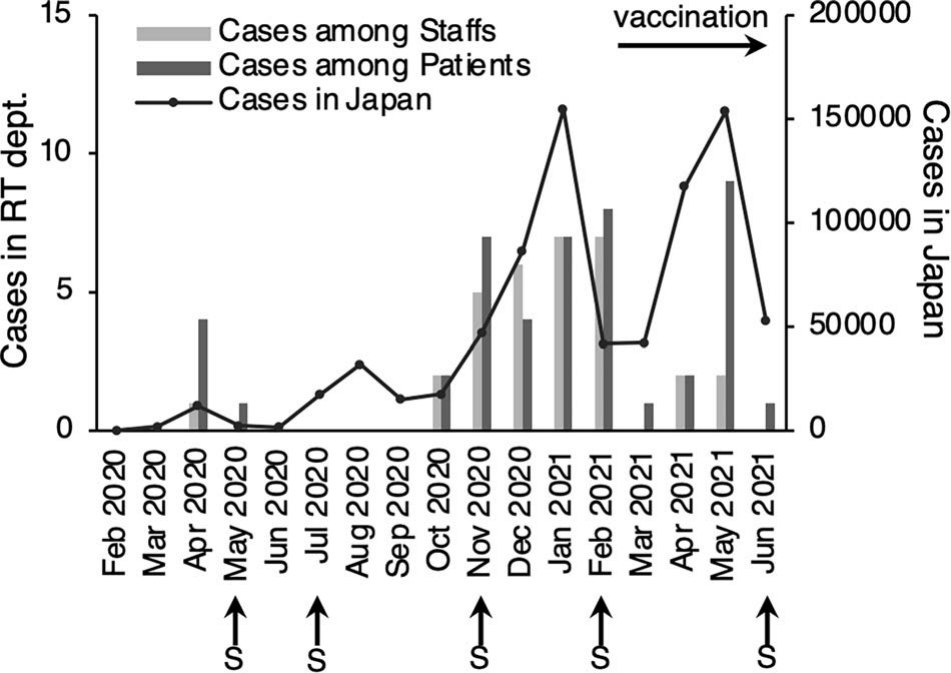
Monthly COVID-19 cases at radiotherapy departments in Japan. The monthly numbers of COVID-19 cases (solid line; right vertical axis) among the patients (dark gray bars) and staff (light gray bars) of radiotherapy departments (bar graph; right vertical axis) in Japan, as reported by the Ministry of Health, Labour and Welfare. The horizontal arrow indicates the prior initiation of COVID-19 vaccination to medical staffs. The vertical arrow indicates the survey (S). Abbreviations: RT, radiotherapy; dept., department.

There were COVID-19 outbreaks in the radiotherapy departments in 47 institutions, among which patient care was not interrupted by the outbreak in 40 institutions. However, services were shut down in seven institutions (approximately 20%) with varying durations (one institution for 3, 5 and 50 days each and four institutions for 1 day). The increase or decrease in the number of patients and staff with COVID-19 was correlated with the total number of infected patients in Japan at that time (Fig. 1). In total, 32 radiotherapy department staff and 46 patients receiving radiotherapy developed COVID-19. Until April 2021, the number of patients and staff who tested positive for COVID-19 was extremely similar. However, in May 2021, the number of staff with COVID-19 was significantly lower than that of patients.

The efficacy of radiotherapy might be reduced if treatment is interrupted or delayed. Therefore, this had been a cause of concern according to the surveys conducted in February and June 2021, which revealed that 33 institutions did not provide treatments to patients with COVID-19. In 10 institutions, treatment was continued after the patients with COVID-19 were identified. Moreover, treatment was initiated owing to urgency despite COVID-19 positivity in one institution (data not shown). The overwhelming percentage (> 98%) of institutions indicated that they discontinue treatment upon the identification of COVID-19-positive status (data not shown).

## DISCUSSION

The COVID-19 pandemic affected normal radiotherapy services in many countries. Owing to differences in COVID-19 prevalence rates, policies, vaccination rates and treatment options, the impact of COVID-19 on the operations and services in radiotherapy departments could vary by country. For example, in China, the number of patients receiving radiotherapy suddenly decreased in the early stages of the 2020 pandemic, primarily due to lockdowns that made it difficult for patients to avail treatment [3]. Moreover, based on a population-based study conducted in the United Kingdom, the number of radiotherapy courses decreased by 19.9% in April, 6.2% in May and 11.6% in June compared with the corresponding months in 2019, thereby indicating a reduction in patient volume. Interestingly, there was an increase in the use of radiotherapy for esophageal, bladder and rectal cancers, which reflects reduced surgical activity [4]. The European Society for Radiotherapy and Oncology (ESTRO) conducted a survey to assess the impact of the COVID-19 pandemic on European radiotherapy departments in May 2020 and February 2021. The patient volume decreased in 2020 compared with 2019 in 53% institutions. Treatment postponement decreased from 58% in May 2020 to 23% in February 2021. Treatment interruptions were observed in 55% of institutions due to COVID-19 positivity in some patients receiving radiotherapy. Telemedicine was commonly used for follow-up visits [8]. Furthermore, the American Society for Radiation Oncology (ASTRO) recently performed a longitudinal survey, which indicated that patient access to radiotherapy was preserved during the pandemic, and most institutions did not defer or postpone the treatment in early 2021. In addition, telemedicine was commonly used in metropolitan areas [9]. In Japan, some institutions experienced a decrease in the number of patients receiving radiotherapy, and the number of patients was limited in the early stages of the pandemic. Nearly all institutions had stopped postponing the start of radiotherapy in June 2021. However, 25–30% of the institutions experienced a decrease in the number of patients compared with the pre-pandemic numbers. There was no severe lockdown in Japan, and COVID-19 was not substantially prevalent in the radiotherapy departments. Hence, the decrease in the number of patients might be attributed to a decrease in new patient consults at the radiotherapy department. Several studies have suggested that the number of patients with potential undiagnosed cancer might be increasing. According to a survey of hospital-based cancer registry conducted by the National Cancer Center Japan, 1 040 379 patients were diagnosed with cancer in 2020, a decrease of 60 409 from 2019 [10]. A previous study in Japan examined the cancer stage at diagnosis in 5167 patients newly diagnosed with gastrointestinal cancers over a 4-year period from 2017 to 2020. Comparing the pre-COVID-19 period with the COVID-19 period, the number of new colorectal cancer diagnoses decreased significantly by 32.9% for Stage 0, 34% for Stage I and 35.3% for Stage II, while it increased significantly by 68.4% for Stage III [11]. Furthermore, the ESTRO [8] and ASTRO [9] surveys outlined the problem of high rates of advanced-stage cancers during the pandemic, which might become an issue in oncology in the future. The workload on radiotherapy departments may increase with an increase in the number of patients with advanced-stage cancer.

Hypofractionated radiotherapy may be useful in reducing the number of patient visits and the workload of radiotherapy departments during a pandemic. Using data from randomized controlled trials, a previous report compared the efficacy of conventional fractionated radiotherapy with hypofractionated radiotherapy and reported that hypofractionation was particularly effective in cases of severe infection [12]. A population-based study conducted in the United Kingdom reported a significant increase in the adoption of ultra-hypofractionated regimen at a dose of 26 Gy in 5 fractions for breast cancer treatment in April 2020, which contributed to reduced patient attendances [4]. Our survey results showed that the hypofractionated regimen for the treatment of breast and prostate cancers as well as for palliation was used more frequently during the pandemic than during the pre-pandemic period. Our survey was not designed to assess the efficacy of hypofractionated regimens for preventing COVID-19; however, it might have helped by reducing the burden on staff during the pandemic.

COVID-19 outbreaks occurred in 47 radiotherapy departments. In May 2021, there was a higher decrease in the number of COVID-19 cases in staff than in patients, which may be attributed to the effect of prior vaccination of healthcare workers from February 17, 2021 onward. Increased vaccination can reduce the incidence of COVID-19 in patients receiving radiotherapy. However, the situation should be cautiously monitored because knowledge on the efficacy of the COVID-19 vaccine in patients with cancer is limited. The SOAP-2 trial investigated the efficacy of the COVID-19 vaccine (Pfizer-BioNTech) in patients with cancer, and the results indicated that that two doses are effective for solid tumors and may be ineffective for hematologic cancers [13]. Only few institutions offered treatment as scheduled in patients undergoing radiotherapy or those who tested positive for COVID-19 and needed to start radiotherapy. Because prolonged overall treatment time in radiotherapy might reduce its efficacy [14], it is recommended that treatment be continued with appropriate infection control measures.

This study had several limitations. First, it had a relatively small sample size. Of 769 radiotherapy institutions that currently operate in Japan according to the Directory of Radiotherapy Centres database [15], we were only able to collect data from 26.1–70.9% institutions based on the survey. Our data might not have represented the national practices. ASTRO and ESTRO surveys have also encountered issues such as low response rates (17.8–25.7% and 21–28%, respectively) [8,9]. The response rate (26.1–70.9%) in our survey was relatively higher than that of these surveys. Second, the institutions participating in the five surveys were not consistently similar. However, despite these limitations, this is a unique report on the impact of the COVID-19 pandemic on radiotherapy departments in Japan with a long survey period.

In conclusion, we surveyed 26.1–70.9% of radiotherapy departments in Japan from May 2020 to June 2021 to assess the impact of the COVID-19 pandemic on their operations. Almost all radiotherapy departments continued to treat patients during the pandemic. However, several institutions reported a significant decrease in the number of patients over time. Although the comprehensiveness of infection control measures among patients and staff has been maintained, some patients receiving radiotherapy presented with COVID-19 infection in approximately 20% of the respondent institutions. Compared to the pre-pandemic period, the hypofractionated regimen was more commonly adopted for the treatment of breast and prostate cancers as well as for palliation during the pandemic. Further follow-up and analysis should be conducted to better understand the long-term impact of COVID-19 on radiotherapy in Japan.

## PRESENTATION AT A CONFERENCE

This study was partially presented in The 33^rd^ annual meeting of Japanese Society for Radiation Oncology on October 1st, 2020, and the 57^th^ Autumn Assembly of the Japan Radiological Society on September 19th, 2021.
